# Identification of a cullin5-RING E3 ligase transcriptome signature in glioblastoma multiforme

**DOI:** 10.18632/aging.103737

**Published:** 2020-09-14

**Authors:** Shuhua Zheng, Zhenhao Li

**Affiliations:** 1 Nova Southeastern University, College of Osteopathic Medicine, Fort Lauderdale, FL 33134, USA; 2 Zhejiang University, College of Pharmaceutical Science, Zhejiang Province 310027, PR China; 3 Zhejiang Key Agricultural Enterprise Institute of Shouxiangu Rare Herb Product, Zhejiang Province 310027, PR China

**Keywords:** glioblastoma multiforme, cullin5-RING E3 ligase, prognosis, SOCS3, neddylation

## Abstract

Glioblastoma multiforme (GBM) is the deadliest type of brain tumor. The median survival time for patients with GBM is only 15 months, even following maximal surgical resection and chemotherapy and radiation therapy. A genetic biomarker could enable a paradigm shift in precise diagnosis, personalized therapeutics and prognosis. In this study, we employed the Chinese Glioma Genome Atlas, The Cancer Genome Atlas, and the Ivy Glioblastoma Atlas Project databases for RNA sequencing (RNA-seq) analysis and clinicopathological studies. We demonstrated that elevated expression of the *RNF7,* *TCEB1*, *SOCS1* and *SOCS3* genes, which encode components of cullin5-RING E3 ligase (CRL5), predict unfavorable GBM prognoses. In GBM and glioma cases carrying *IDH1* mutations, *SOCS1* and *SOCS3* methylation was increased and their expression was downregulated. This study has thus identified a simple transcriptome signature for GBM prognosis.

## INTRODUCTION

Glioblastoma multiforme (GBM) is the most common and aggressive tumor of the central nervous system (CNS) in adults [1, 2]. The Central Brain Tumor Registry of the United States reports that the average annual incidence rate of malignant CNS tumors in the United States is 23.41 per 100,000, 48.3% of which are GBM [3]. Rigorous screening algorithms have been used to process genome-wide databases, revealing multiple prognostic transcriptome signatures with no apparent functional interplay [4–9]. Many recent pre-clinical studies have identified the cullin-RING E3 ligase (CRL) complex in the ubiquitin-proteasomal system (UPS) as a promising target for GBM treatment [10–12].

**Figure 1:**
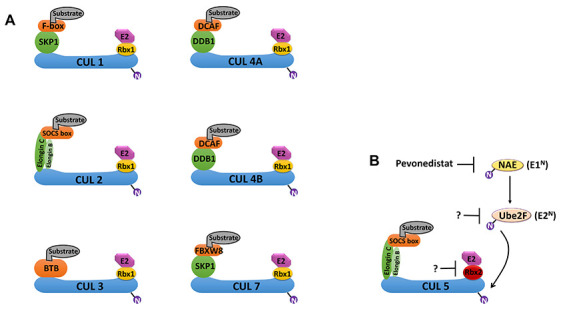
**Schematic overview of cullin-RING E3 ligases (CRLs) and the NEDD8 conjugation pathway.** (**A**) Cullin (CUL) proteins form the scaffold of the CRL E3 ligase complexes. CRL1 and CRL7 use SKP1; CRL2 and CRL5 use both Elongin B and Elongin C; CRL4A and CRL4B use DDB1; and CRL3 uses BTB as substrate adaptors. CRL1 uses F-box proteins; CRL4A and CRL4B use DCAF; CRL2 and CRL5 use SOCSs; and CRL7 uses FBXW8 as substrate binding proteins. CRL1-3, 4A/B and 7 use Rbx1; and CRL5 uses Rbx2 as RING-finger proteins. (**B**) Conjugation of the Ub-like protein NEDD8 to a cullin protein is required for fully activation of the CRL. The conjugation of NEDD8 occurs in three steps: activation by the NEDD8 activation enzyme (NAE; E1^N^), transference to the E2 enzyme (E2^N^), and conjugation of NEDD8 to the cullin protein in the CRL. UBE2F is the major E2^N^ needed for NEDD8 conjugation to CRL5. MLN4924 (Pevonedistat) is a first-in-class inhibitor of E1^N^ that can prevent NEDD8 conjugation to CRLs. Abbreviations: Ub, ubiquitin; RING, really interesting new gene; DDB1, DNA damage-binding protein 1; DCAF, DDB1- and CUL4-associated factor; FBXW8, F-box and WD40 domain 8; BTB, broad-complex, tramtrack, and bric-à-brac; SOCS, suppressor of cytokine signaling.

Conjugation of ubiquitin (Ub) in the UPS is achieved through a cascade of three consecutive enzymatic reactions catalyzed by the E1, E2 and E3 enzymes. The E1 ubiquitin-activating enzyme (UAE) activates Ub and catalyzes the covalent transfer of Ub to the E2 Ub-conjugating enzyme. An E3 ligase catalyzes the final step by transferring Ub from E2 to a substrate protein [13]. CRLs polyubiquitinate approximately 20% of cellular proteins degraded *via* the UPS [13]. The cullin protein forms a central stalk-like scaffold that constrains and positions the substrate binding and adaptor proteins on its N-terminus and the RING-finger containing proteins (Rbx1 or Rbx2) on its C-terminus (Figure 1) [14]. The catalytic centers of CRLs label substrates with a chain of Ubs, earmarking those proteins for proteasome-mediated degradation [14]. Malignant cells often display CRL overactivation, which promotes cell-cycle progression, signaling pathway dysregulation and chemo-/radioresistance [15, 16]. CRL activation requires conjugation of a Ub-like protein called neural precursor cell-expressed developmentally downregulated 8 (NEDD8) near the C-terminus of cullin [17]. NEDD8 conjugation to cullin is also carried out in three stepwise enzymatic reactions. These reactions involve the NEDD8-activating enzyme (NAE; E1^N^), UBC12 and UBE2F (E2^N^) and E3^N^, which transfers NEDD8 to cullin (Figure 1) [17]. Hyperactivation of NEDD8 conjugation predicts poor GBM prognosis [12]. Human cells have 7 different cullin proteins (CUL1, 2, 3, 4A, 4B, 5, 7), and each cullin protein has a corresponding substrate recognition protein, an adaptor protein and a RING-finger containing protein (Figure 1).

We report here that the expression of the *RNF7,* *TCEB1*, *SOCS1* and *SOCS3* genes, which encode the RING-finger protein Rbx2, the adaptor protein Elongin C and the substrate binding proteins SOCS1 and SOCS3, respectively, are simultaneously upregulated in GBM. Rbx2, Elongin C, SOCS1 and SOCS3 form a specific E3 ligase known as CRL5 (Figure 1). Given the importance of CRLs in GBM progression, the transcriptome signature of CRL5 components may have important GBM grading, diagnostic, prognostic and therapeutic value.

## RESULTS

### Anatomically mapped differential gene expression patterns of CRLs

**Figure 2:**
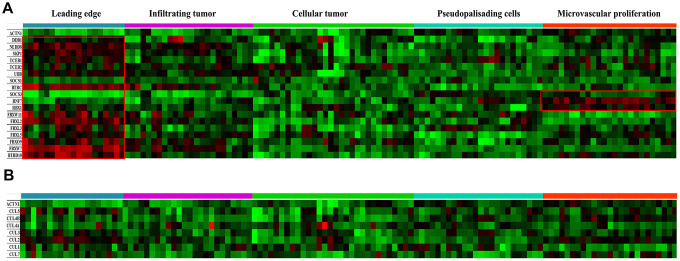
**Anatomical mapping of CRL components in GBM clinical samples.** (**A**) Expression levels of major CRL component genes *SKP1, TCEB1/2, BTBD10, DDB1, TCEB1, TCEB2*, *BTRC, SOCS1, SOCS3, FBXL2/3/5, FBXW7/11, FBXO9*, *RBX1*, *RNF7*, *UBB*, and *NEDD8,* were mapped based on the anatomic features of clinical samples. (**B**) Expression levels of the cullin genes *CUL1, CUL2, CUL3, CUL4A, CUL4B, CUL5* and *CUL7* were also mapped. The following anatomic structures were identified by H&E staining based on the Ivy GAP dataset: leading edge, infiltrating tumor, cellular tumor, microvascular proliferation and pseudopalisading cells around necrosis. *ACTN1* gene expression was used as a reference.

**Figure 3:**
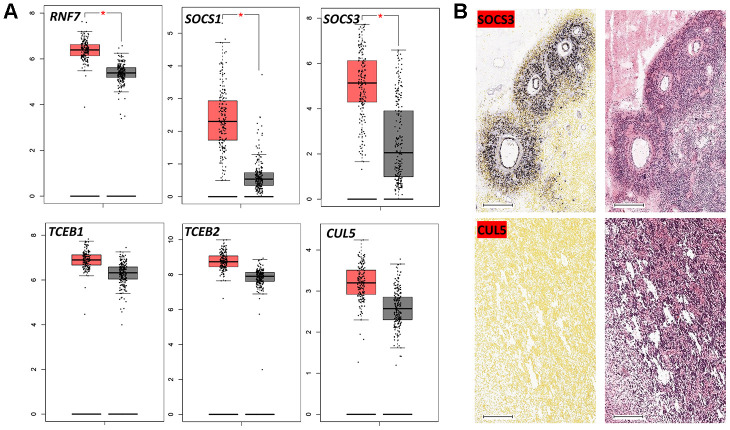
**Expression of CRL5 components in GBM.** (**A**) Boxplot data analysis of genes encoding CRL5 components in GBM and normal brain tissues. GEPIA (http://gepia.cancer-pku.cn/) was used to analyze RNA-seq data from malignant and normal tissues (TCGA and GTEx datasets). (**B**) *In situ* hybridization of *SOCS3* and *CUL5* in GBM patients based on the Ivy GAP dataset. * *P* < 0.01.

The histological dataset from the Ivy Glioblastoma Atlas Project (Ivy GAP) was classified anatomically as leading edge (LE), infiltrating tumor (IT), cellular tumor (CT), pseudopalisading cells around necrosis (PAN) and microvascular proliferation (MVP) based on H&E staining analysis. Expression levels of genes encoding key components of CRLs were mapped in Figure 2A. *SKP1*, *BTBD10*, *DDB1*, *TCEB1* and *TCEB2* genes encode the adaptor proteins; *BTRC, SOCS1, SOCS3, FBXL2/3/5, FBXW7/11* and *FBXO9* genes encode the substrate-binding proteins; *RBX1* and *RNF7* genes encode the RING-finger containing proteins Rbx1 and Rbx2, respectively; and *UBB* and *NEDD8* genes encode Ub and NEDD8, respectively [14]. The expression level of the majority of CRL components was higher at the LE anatomical structure (Figure 2A). The expression levels of *SOCS3*, *RNF7* and *RBX1* were higher in the MVP anatomical structure (Figure 2A). *SOCS1* and genes encoding all cullin proteins were evenly distributed (Figure 2A and 2B). The ISH data confirmed that *SOCS3* expression is higher in perivascular area (Figure 3B). SOCS1 and SOCS3 function in both CRL2 and CRL5 complexes, whereas Rbx2 (encoded by *RNF7*) is unique in that it mainly functions in the CRL5 complex (Figure 1).

### Upregulated expression levels of CRL5 components in GBM

Using the TCGA and GTEx datasets, we compared the differential expression levels of the genes encoding CRL2 and CRL5 in GBM and normal tissues. Expression levels of *RNF7*, *SOCS1* and *SOCS3* were significantly higher in GBM tissues than in normal tissues (Figure 3A). However, we did not observe significantly upregulated expression levels of *CUL2*, *CUL5* or *RBX1* in GBM tissues compared with those in normal tissues (data not shown).

### The correlation between GBM staging and CRL5 expression

**Figure 4:**
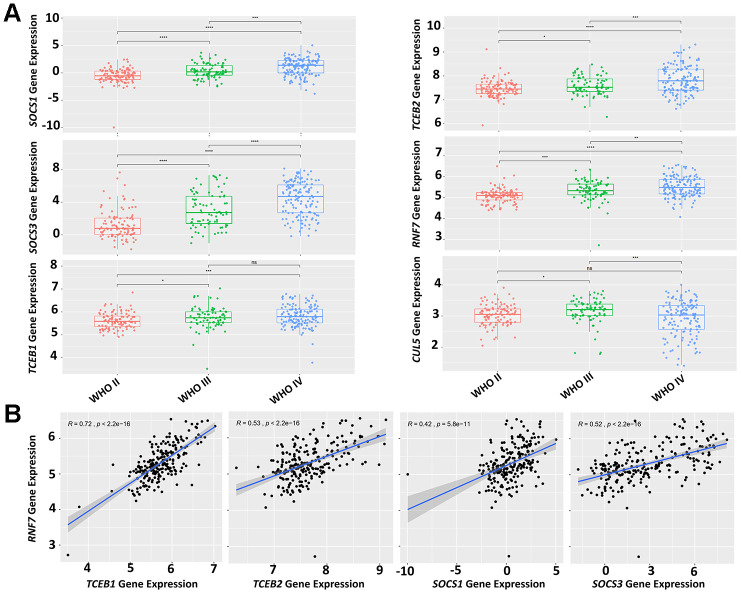
**Expression levels of CRL5 components correlate with glioma grading.** (**A**) A CGGA dataset (mRNAseq_325) was used for analysis of CRL5 component gene expression and glioma grading. (**B**) A CGGA dataset (mRNAseq_325) was used for correlation analysis of gene expression between *RNF7* and *TCEB1*, *TCEB2*, *SOCS1* and *SOCS3* with *R* values of 0.72, 0.53, 0.42, 0.52, respectively. The *P* values in each pair were all less than 1e^-10^.

The WHO classification offers primary guidelines for brain tumor grading and treatment. However, the grading system for glioma largely depends on microscopic histological features, because molecular biomarkers are not available [18]. We determined if the expression of CRL5 components was correlated with WHO tumor classification. In the Chinese Glioma Genome Atlas (CGGA) dataset, we found that elevated expression levels of *SOCS1, SOCS3, TCEB1, TCEB2* and *RNF7* correlated with more advanced WHO grades (grades III and IV) of primary glioma (Figure 4A). The expression levels of *CUL5* did not correlate with glioma WHO grades (Figure 4A). We further studied whether the SOCS1, SOCS3, Rbx2, Elongin B and Elongin C proteins form a functional complex. We found that the expression levels of *RNF7* correlate with those of *TCEB1* and *TCEB2*, *SOCS1*, *SOCS3*, with *R* values of 0.72, 0.53, 0.42 and 0.52, respectively (Figure 4B). These results indicate that components of CRL5 form a functional protein complex in GBM, and their gene expression levels can be valuable grading biomarkers.

### Expression levels of CRL5 components as survival predictors

**Figure 5:**
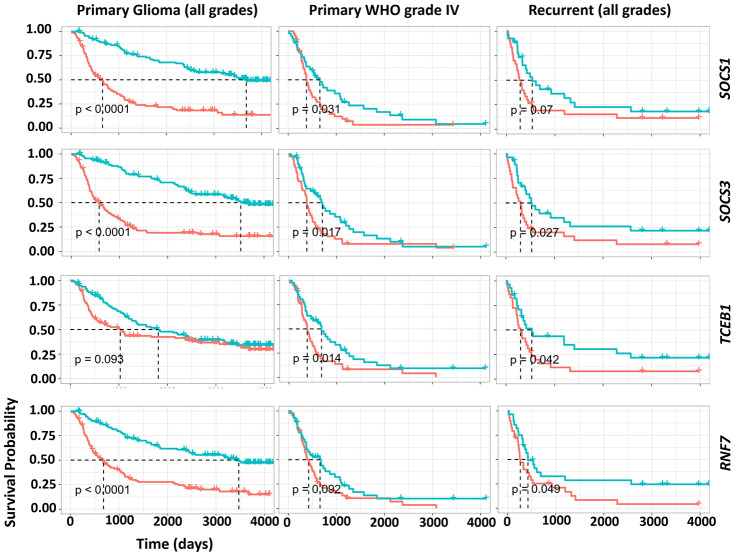
**Impact of gene expression of CRL5 components on GBM, primary and recurrent glioma survival.** The survival analysis of WHO grade IV, primary and recurrent gliomas was based on a CGGA dataset (mRNAseq_325). *P* values are indicated in the picture.

We also studied the potential of CRL5 as a biomarker in predicting overall survival (OS) rates in patients with GBM and glioma. Patients with primary glioma, WHO grade IV glioma or recurrent glioma were grouped into cohorts based on the median expression levels of CRL components (Figure 5). We found that primary glioma patients with higher-than-median expression levels of either *SOCS1*, *SOCS3* or *RNF7* had an unfavorable prognosis compared to those with lower-than-median expression levels (*P* < 0.0001) (Figure 5). A higher-than-median expression level of *SOCS1*, *SOCS3* or *TCEB1* also predicts a poor prognosis for WHO grade-IV glioma patients compared to those with lower-than-median expression (Figure 5). (*P* values were 0.031, 0.017 and 0.014, respectively). Patients with primary glioma, WHO grade IV glioma, and recurrent glioma in whom *SOCS3* expression levels were higher than the median consistently demonstrated unfavorable OS rates compared with those with lower-than-median *SOCS3* expression levels (Figure 5). (*P* values were < 0.0001, 0.027 and 0.017, respectively.) However, *TCEB2* expression levels failed to predict WHO grade IV glioma prognosis (data not shown).

### A GBM prognosis index (PI) system based on a four-gene signature

**Figure 6:**
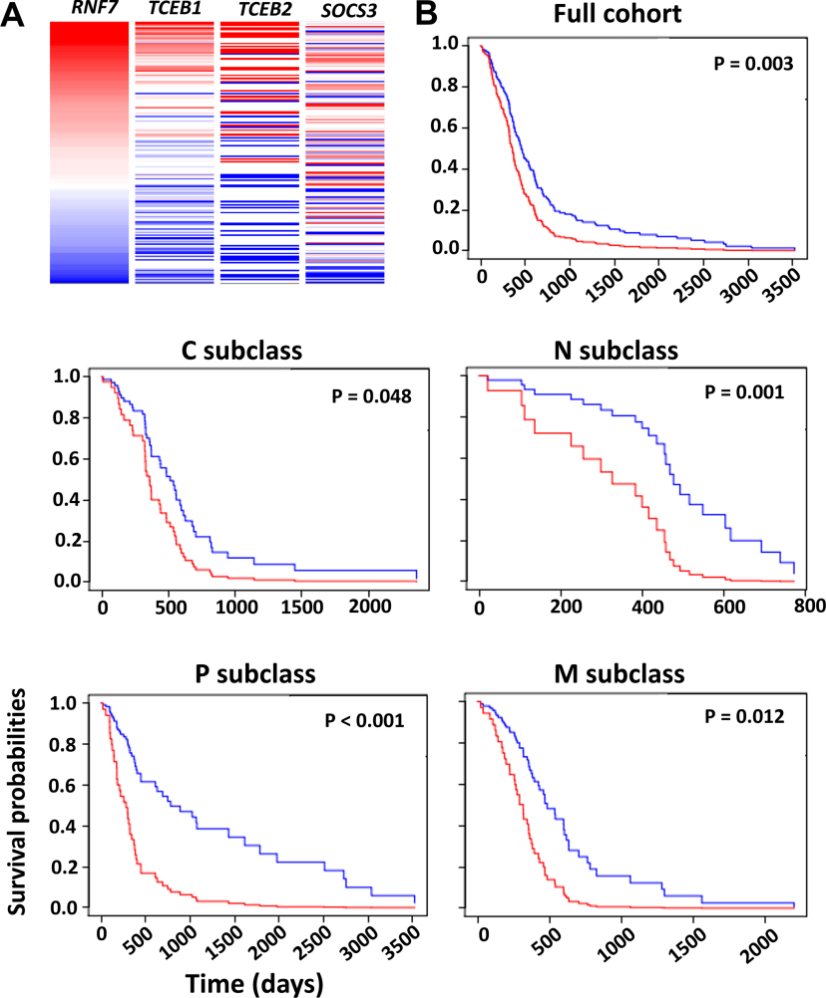
**Survival analysis based on the impact of the multi-gene PI.** (**A**) Heatmap analysis of the CRL5 components in TCGA-GBM dataset was conducted using the UCSC Xena tool. (**B**) Survival analysis based on HR was conducted using the GBM-BioDP portal. GBM tissue was classified into proneural (P), neural (N), classical (C) and mesenchymal (M) subtypes based on gene expression patterns [20]. A Cox proportional hazards model was constructed with gene expression of the CRL5 components, *SOCS3*, *RNF7*, *TCEB1* and *TCEB2*. We used O-6-methylguanine-DNA methyltransferase (*MGMT*) methylation status as a covariate. *P* values are included in the figure.

Glioma patients harboring an *IDHs* mutation and 1p/19q co-deletion often have favorable clinical outcomes [19]. Both SOCS1 and SOCS3 in the CRL5 function as substrate-binding proteins that recruit target proteins for Ub conjugation. Due to low basal expression levels of *SOCS1* in GBM and the gene’s potential functional overlap with *SOCS3*, we decided to exclude *SOCS1* in our multi-gene analysis for GBM prognosis. *SOCS3*, *RNF7*, *TCEB1* and *TCEB2* are genes encoding key components of the CRL5 complex. These genes were first analyzed on the UCSC Xena tool using an RNA-seq dataset of 172 The Cancer Genome Atlas Glioblastoma Multiforme (TCGA-GBM) patients (https://xena.ucsc.edu/) (Figure 6A). The heatmap analysis confirmed a linear correlation between the selected genes, and a Cox model was warranted to calculate the PI, also known as the risk score (Figure 6A). Verhaak et al. classified GBM into the following four types: classical, mesenchymal, proneural and neural (C, M, P, N) [20]. Using O-6-methylguanine-DNA methyltransferase (*MGMT*) gene methylation status as a covariate, we evaluated the PI for the four-gene RNA-seq signature in all GBM subclasses of a GBM-BioDP dataset. We found that the four-gene signature had PI values of 1.61, 1.86, 2.71, 3.66 and 3.45 for the full GBM cohort, and the C, M, P and N subclasses, respectively (Figure 6B). Hazard ratio (HR)-based *P*-value analysis yielded *P* values of 0.003, 0.048, 0.001, < 0.001 and 0.012 for the full GBM cohort, and the C, M, P and N subclasses, respectively (Figure 6B). These data indicate that the four-gene signature encoding the CRL5 components Elongin B, Elongin C, SOCS3 and Rbx2 can predict the OS of all the GBM subclasses.

### Correlation of *SOCS1* and *SOCS3* expression with *IDHs* mutations

**Figure 7:**
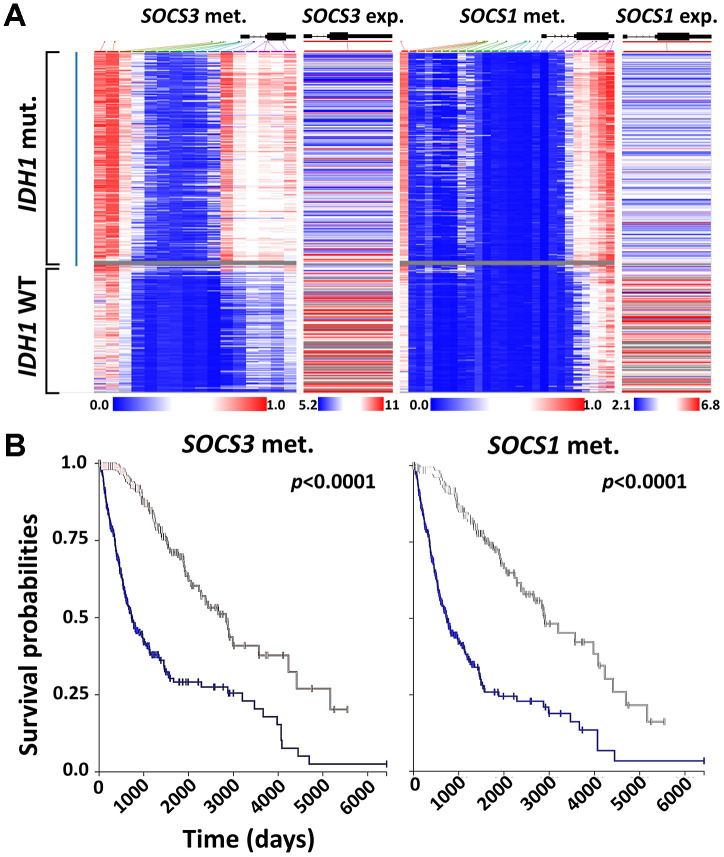
**Correlation between *SOCS1* and *SOCS3* expression and *IDH1* mutation.** (**A**) Heatmap analysis of *SOCS1* and *SOCS3* gene expression, methylation and *IDH1* mutation status was achieved using UCSC Xena and TCGA-LGG/GBM datasets (N = 658). The gray area indicates that *SOCS3* methylation data is not available for that specific patient’s sample. (**B**) K-M survival analysis of LGG and GBM patients based on the methylation status of *SOCS1* and *SOCS3*. Blue lines indicate the groups with lower-than-median methylation of *SOCS1* and *SOCS3*.

We demonstrated that elevated expression levels of either *SOCS1* or *SOCS3* could independently predict poor prognosis of patients with primary glioma or GBM. Recent studies have found that *SOCS1* and *SOCS3* genes are hypermethylated in various types of cancers [21, 22]. We conducted a heatmap analysis of a cohort of 658 patients in TCGA Low-Grade Glioma and GBM (TCGA-LGG/GBM) dataset (Figure 7A). We found that *IDH1* mutation predicts hypermethylation and expression downregulation of *SOCS1* and *SOCS3* (Figure 7A). Furthermore, patients with hypermethylation of either *SOCS1* or *SOCS3* have favorable OS rates regardless of treatment regimens (Figure 7B). To study the potential correlation between *IDH1* mutation status and *SOCS1*/*SOCS3* expression levels, we identified 7 patients carrying missense/in-frame *IDH1* mutations whose *SOCS3* expression levels were also available in the TCGA-GBM dataset. All 7 patients with mutated *IDH1* had lower-than-median *SOCS3* expression levels and promoter hyperme thylation (Supplementary Figure 1). We found that *SOCS1* expression levels are not closely related to methylation status in TCGA-GBM dataset (data not shown). Together these data indicate that *IDH1* mutations may cause *SOCS1* and *SOCS3* hyperme thylation and expression downregulation.

## DISCUSSION

In this study, we demonstrated that overexpression of the CRL5 components Elongin B, Elongin C, SOCS3 and Rbx2 predicts poor prognosis of glioma and all GBM subclasses. We also found that elevated expression levels of *TCEB1*, *TCEB2*, *SOCS3* or *RNF7* correlate with higher glioma grades, providing new molecular parameters for glioma grading. Furthermore, we showed that the transcription of *SOCS1* and *SOCS3* is downregulated in *IDH1*-mutated glioma cases *via* methylation, suggesting *IDHs* mutations can regulate protein turnover [23]. SOCS1 and SOCS3 mediate degradation of the NF-κB family member p65/RelA, focal adhesion kinase (FAK), von Hippel Lindau (vHL), Janus kinase 2 (JAK2) and indoleamine 2,3-dioxygenase (IDO), all of which play essential roles in GBM progression and chemo-/radioresistance (Table 1) [24–28]. We also employed proteomic studies to show that elevated protein levels of cullin5 were inversely correlated with those of vHL and p65/RelA (Supplementary Figure 2A). We found that CRL5-mediated anti-angiogenic vHL protein downregulation may promote GBM neovascularization, as indicated in our immunohistochemical studies. GBM samples with high-intensity cullin5 staining had more enriched vasculature than those with low cullin5 immunoreactivity (Supplementary Figure 2B). However, upregulated CRL5 activity also mediated degradation of p65/RelA, an important oncogenic transcriptional factor for GBM progression [27].

**Table 1:** Involvement of cullin5-RING E3 ligase (CRL5) in GBM progression.

**Key Substrates**	**Pathways**	**Major functions in GBM**
von Hippel–Lindau	VHL/HIF oxygen-sensing	Angiogenesis; invasion; radio-resistance
p65/RelA	NF-κB pathway	Chemo-/radio-resistance
Focal adhesion kinase	Cell adhesion signaling	Chemo-/radio-resistance
Janus kinase 2	JAK/STAT signaling	Chemo-/radio-resistance
Indoleamine 2,3-dioxygenase	IDO pathway	Immune evasion

VHL, p65/RelA, FAK, JAK2 and IDO are CRL5 substrates. VHL is involved in GBM angiogenesis, invasion and radio-resistance [39]. p65/RelA, FAK and JAK2 are critical in mediating GBM chemo-/radio-resistance [26, 40]. IDO is mainly involved in GBM immune evasion [25]. Abbreviations: FAK, focal adhesion kinase; JAK2, Janus kinase 2; IDO, indoleamine 2,3-dioxygenase; STAT, signal transducer and activator of transcription; vHL, von Hippel-Lindau.

CRL5-independent activities of SOCS3 involve mitogen-activated protein kinase (MAPK) pathway activation, as well as JAK-signal transducer and activator of transcription (STAT) pathway inhibition [29, 30]. Our gene expression heatmap analysis showed that *SOCS3* expression levels correlate poorly with those of *RNF7*, *TCEB1* and *TCEB2* in GBM samples. Also, *SOCS3* expression was clustered around capillaries, whereas *CUL5* expression was evenly distributed (Figures 2A and 3B). This pattern of distribution is consistent with previous studies suggesting the upregulation of *SOCS3* transcription levels in areas with higher O_2_ saturation [29].

Our study suggests that strategies targeting CRL5 may curb GBM progression. CRL5 activation requires conjugation of a Ub-like small peptide called NEDD8 at the C-terminus of cullin5. This conjugation is mediated by the coordinated action of enzymes such as NAE and E2^N^ (Figure 1). Inhibition of NAE using the first-in-class inhibitor MLN4924 can downregulate CRL5 activity. However, ubiquitination of oncogenic proteins such as HIF-1α, β-catenin and Mcl-1 is also carried out by CRLs. Therefore, inhibition of NEDD8 conjugation will lead to the stabilization of these oncogenic substrates, highlighting the necessity of developing CRL5-specific inhibitors for potential therapeutic management of GBM [31–36]. Unfortunately, due to a lack of druggable pockets, CRL complexes are difficult to target pharmaceutically [37]. Two E2^N^s, UBC12 (also known as UBE2M) and UBE2F, are found in mammalian cells. UBC12 interacts with Rbx1 to mediate the neddylation of cullin1-4, and UBE2F pairs with Rbx2 to facilitate the neddylation of cullin5 [38]. Therefore, inhibition of UBE2F and Rbx2 will eradicate NEDD8 conjugation to cullin5, offering a strategy to specifically target CRL5 without interfering with other CRLs (Figure 1).

## MATERIALS AND METHODS

### Public datasets

The primary databases of samples were derived from The Cancer Genome Atlas Glioblastoma Multiforme (TCGA-GBM) and the Chinese Glioma Genome Atlas Network (CGGA) [41]. The primary clinicopathological samples with anatomic transcriptional atlases were derived from the Ivy Glioblastoma Atlas Project (Ivy GAP) [42]. All samples were collected with informed consent. TCGA-GBM provided information from 206 GBM patients on copy number variation, RNA-seq and DNA methylation. The CGGA dataset provided grading, RNA-seq and DNA methylation information from 1,962 glioma patients. The TCGA Low-Grade Glioma and GBM (TCGA-LGG/GBM) dataset provided information from 1,153 patients on DNA methylation, RNA-seq and isocitrate dehydrogenases (*IDHs*). Expression levels of genes in normal tissue were derived from the Genotype-Tissue Expression (GTEx) project (https://www.gtexportal.org/home/).

### Anatomical mapping of CRL components

The Ivy GAP dataset has a 41-patient cohort whose tumor samples were evaluated based on anatomic features classified as leading edge (LE), infiltrating tumor (IT), cellular tumor (CT), pseudopalisading cells around necrosis (PAN) and microvascular proliferation (MVP). Classification of these anatomic features was carried out using hematoxylin and eosin (H&E) staining. Genes encoding major CRLs components including *SKP1*, *BTBD10*, *DDB1*, *TCEB1*, *TCEB2*, *BTRC*, *SOCS1*, *SOCS3*, *FBXL2/3/5*, *FBXW7/11*, *FBXO9*, *RBX1*, *RNF7*, *UBB*, *NEDD8*, *CUL1*, *CUL2*, *CUL3*, *CUL4A*, *CUL4B*, *CUL5* and *CUL7* were included. *ACTN1* gene expression was used as a reference. *In situ* hybridization (ISH) for *CUL5* and *SOCS3* were also derived from the Ivy GAP dataset.

### Kaplan-Meier(K-M) survival analysis

K-M survival analyses for primary glioma of all grades, World Health Organization (WHO) grade IV and recurrent glioma were derived from the CGGA data (mRNAseq_325). Cohorts with sample sizes of 224, 85 and 57 were included for overall survival (OS) analysis of primary, WHO grade IV and recurrent gliomas. Median expression levels of the *SOCS1*, *SOCS3*, *RNF7*, *TCEB1* and *TCEB2* genes were used as cutoffs for classification of patients with lower-than-median and higher-than-median expression levels in each group of analysis. A log-rank test was used for calculations of *P* values.

### Immunohistochemistry (IHC) and proteomic analysis

The Human Protein Atlas provides immunehistochemical analysis of cullin5 expression in GBM samples (https://www.proteinatlas.org/). The IHC study was carried out using anti-cullin5 antibody (HPA002185) Sigma-Aldrich (MO, USA). The antibody reliability is validated for IHC study by assessing staining pattern in 44 normal tissues (https://www.proteinatlas.org/ENSG00000166266-CUL5/antibody). A total of 6 GBM cases were included in the study. Proteomic data used in this publication was generated by the National Cancer Institute Clinical Proteomic Tumor Analysis Consortium (https://cptac-data-portal.georgetown.edu/) (N = 99). Mass spectrometry analysis was conducted using the 11-plexed isobaric tandem mass tags (TMT-11). Protein abundance was presented as log2-ratio of the expression of the sample to a normal control. GBM cases were aligned with decreasing cullin5 protein levels. Corresponding levels of von Hippel Lindau (vHL), p65/RelA, focal adhesion kinase (FAK) and Janus kinase 2 (JAK2) proteins from those aligned cases were color-coded in the heatmap analysis. The color red represents the samples with the 20% with the highest protein levels and color green represents those the 20% with the lowest protein levels.

### Multi-gene prognostic index analysis

Survival analysis based on the impact of the multi-gene prognostic index (PI) was conducted on the GBM-BioDP website (https://gbm-biodp.nci.nih.gov/) using the Verhaak 840 Core experiment setting [20]. Briefly, GBM was classified into proneural (P), neural (N), classical (C) and mesenchymal (M) subtypes based on gene expression patterns [20]. A Cox proportional hazards model was constructed using the gene expression levels of *SOCS3*, *RNF7*, *TCEB1*, *TCEB2* and O-6-methylguanine-DNA methyltransferase (*MGMT*) methylation status as covariates.

### Statistical analysis

Statistical analysis was performed using GraphPad Prism 6.0 software. Pearson correlation analysis between *RNF7* and *TCEB1,* *TCEB2*, *SOCS1* and *SOCS3* expression levels in primary glioma was carried out automatically in the CGGA dataset. All statistical tests were 2-sided, and *P* values less than 0.05 were considered statistically significant.

## Supplementary Material

Supplementary Figures
