# Supplementary material for: Identification of a cullin5-RING E3 ligase transcriptome signature in glioblastoma multiforme
Source: Aging (Albany NY). 2020 Sep 14;12(17):17380–92. doi: 10.18632/aging.103737 (PMC7521521; doi:10.18632/aging.103737)
Supplement: Supplementary Figures [file aging-12-103737-s001..pdf]

SUPPLEMENTARY FIGURES

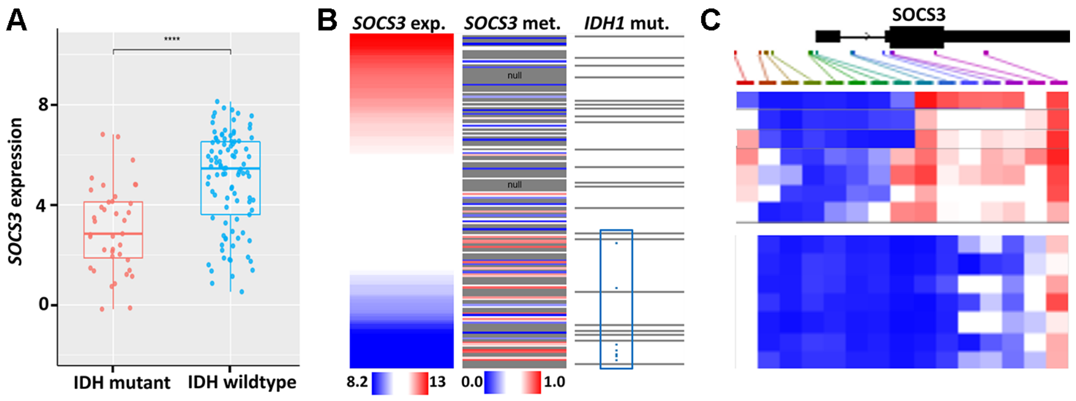

Supplementary Figure 1. *SOCS3* expression and *IDH1* mutations in GBM. (**A**) Boxplot analysis of *SOCS3* expression in *IDH* mutant and wildtype glioma samples was based on CGGA dataset (mRNAseq_325). (**B**) Heatmap analysis of *SOCS3* gene expression and methylation, and *IDH1* mutation status was based on UCSC Xena based on TCGA-GBM dataset. The blue box encompasses all the patient's samples (n=7) that carry mutated *IDH1* with *SOCS3* expression data available (n=166). (**C**) 7 out of 16 *IDH1* mutant patients have *SOCS3* methylation data available (top panel). *SOCS3* methylation status of 7 random GBM patients with unknown *SOCS3* expressions (bottom panel). Gray area indicates *SOCS3* methylation data is not available for that specific patient's sample.

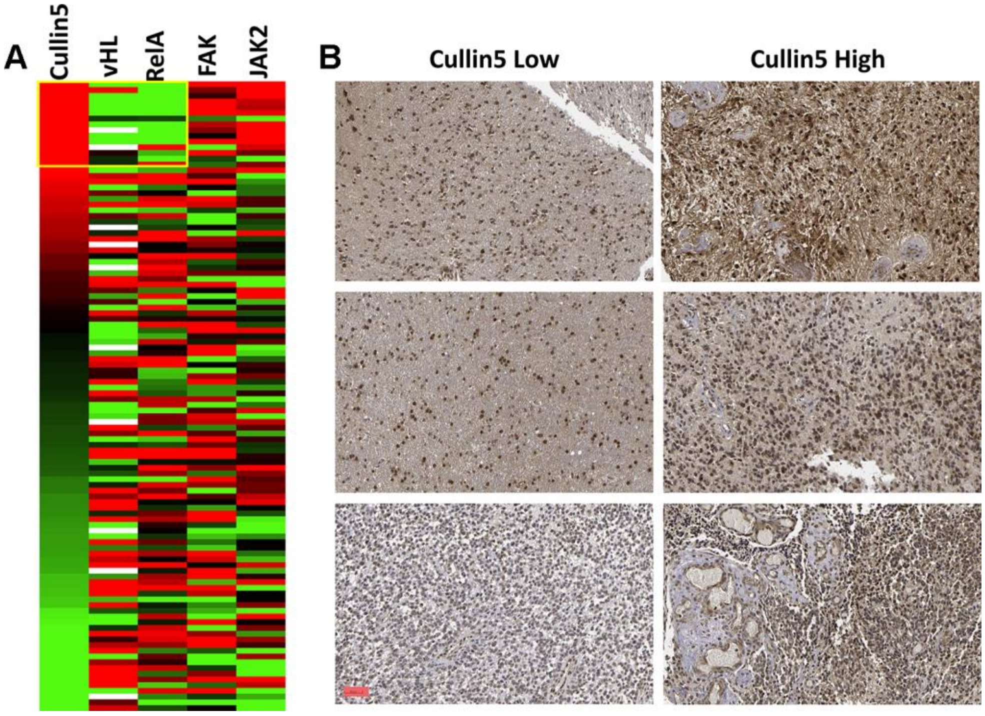

Supplementary Figure 2. Proteomic and immunohistochemistry (IHC) study of cullin5 (**A**) The protein quantification data was derived from CPTAC-GBM dataset (n = 99). GBM cases were aligned with decreasing cullin5 protein levels. Corresponding protein levels of vHL, RelA, FAK and JAK2 of those aligned cases were color coded. (**B**) Presentative cullin5 immunostaining slides derived from 6 HGG patients. Magnification scale: 50 µm. Image credit: Human Protein Atlas. Images available from v19.proteinatlas.org.
